# Coronavirus disease 2019 and catastrophic antiphospholipid syndrome: Case report

**DOI:** 10.1097/MD.0000000000041790

**Published:** 2025-03-28

**Authors:** Shanshan Jin, Shiquan Wu, Bin Cai, Jian Luo

**Affiliations:** a Physical Examination Department, Quzhou Central Blood Station, Quzhou, Zhejiang Province, China; b Department of Critical Care Medicine, The Quzhou Affiliated Hospital of Wenzhou Medical University, Quzhou People’s Hospital, Quzhou, Zhejiang Province, China.

**Keywords:** case report, catastrophic antiphospholipid syndrome, COVID-19, IgA anti-β2-glycoprotein I antibody, multiple thrombi

## Abstract

**Rationale:**

The emergence of catastrophic antiphospholipid syndrome (CAPS) alongside coronavirus disease 2019 (COVID-19) is of great concern, because of its high mortality and unclear mechanism. This severe disease, characterized by multiple thrombi and multisystem disorder, has notably diverse clinical presentations, which complicates its diagnosis in clinical practice. Now, we report a rare case of CAPS in a patient with COVID-19.

**Patient concerns:**

A 64-year-old patient who mainly presented with pain and swelling 2 months ago progressed gradually into multiple thrombi, including pulmonary embolism, renal embolism, and deep vein thrombosis; transient ischemic attack; multiple organ dysfunction with acute kidney injury; and necrosis of both lower limbs, left upper extremity, both ears, and penile gangrene.

**Diagnoses:**

He was diagnosed as CAPS with COVID-19 by positive severe acute respiratory syndrome coronavirus-2 (SARS-CoV-2) testing and high-titer immunoglobulin (Ig) A anti-β2-glycoprotein I antibody (anti-β2GPI).

**Interventions:**

Active rescue treatments such as anticoagulants, plasmapheresis, glucocorticoid pulse therapy, antibiotics, and multi-organ functional support alleviated the disease effectively.

**Outcomes:**

Although his clinical symptoms were successfully controlled, we could not save the necrotic tissue. The patient refused to undergo limb amputation and died of necrotic tissue infection.

**Lessons:**

CAPS in patients with COVID-19 is an extremely serious disease with a high mortality rate. A delay in diagnosis and treatment can result in potentially devastating consequences. Therefore, physicians should be alert to the possibility of CAPS in patients with multiple thrombi and COVID-19. Furthermore, this case serves as a foundation upon which future studies can build to investigate the possible mechanisms of IgA anti-β2GPI-positive CAPS in patients with COVID-19, which may guide the exploration of potential therapeutic strategies to prevent the disease’s progression.

## 1. Introduction

Since its outbreak in 2019, coronavirus disease 2019 (COVID-19), caused by severe acute respiratory syndrome coronavirus-2 (SARS-CoV-2), has become a pandemic. Coagulation disorders and thrombosis, characterized by arterial, venous, and microvascular thromboses, occur frequently in patients with COVID-19.^[1]^ Antiphospholipid antibodies (aPL), which are conventionally positive in antiphospholipid syndrome (APS) and its severe form, catastrophic antiphospholipid syndrome (CAPS), are consistently reported in these patients.^[2]^ The occurrence of multiple thrombi and multi-organ failure in CAPS and COVID-19 may share common pathogenic mechanisms. Non-criteria aPL, such as immunoglobulin (Ig) A anti-β2-glycoprotein I antibody (anti-β2GPI), which have promising clinical implications, have been proposed as additional indicators, especially in seronegative APS (SNAPS).^[3]^ This report describes a CAPS patient with COVID-19 who presented with pain and swelling caused by multiple thrombi and multiple organ dysfunction, and was positive for IgA anti-β2GPI on testing.

## 2. Case presentation

**Figure 1.**
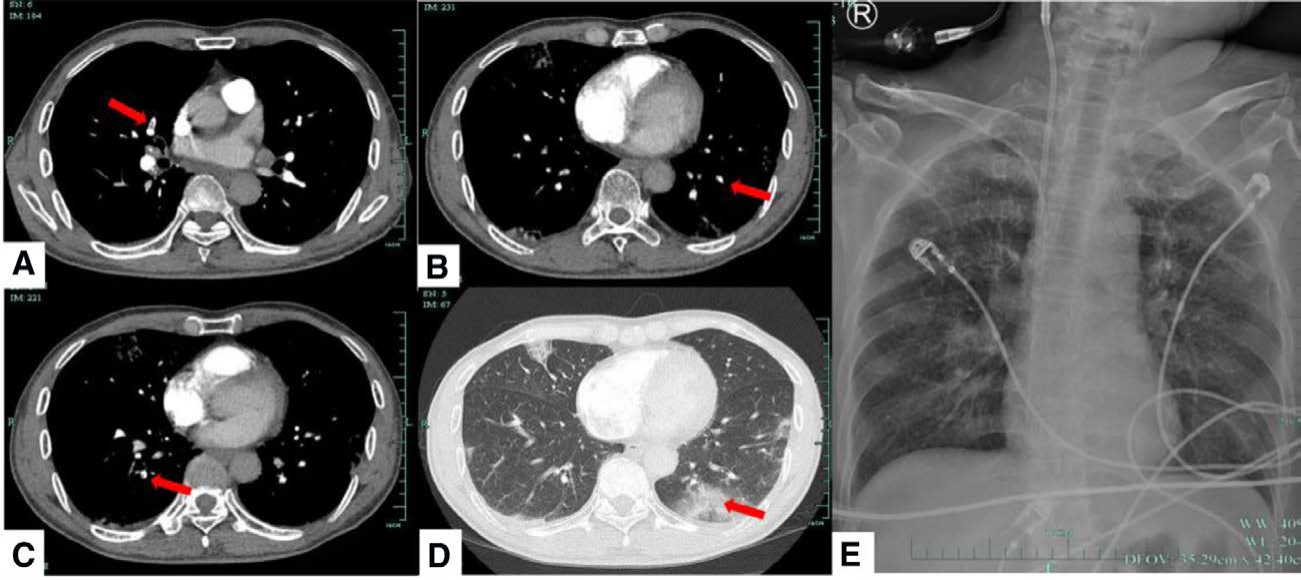
Pulmonary imaging of the patient.

**Figure 2.**
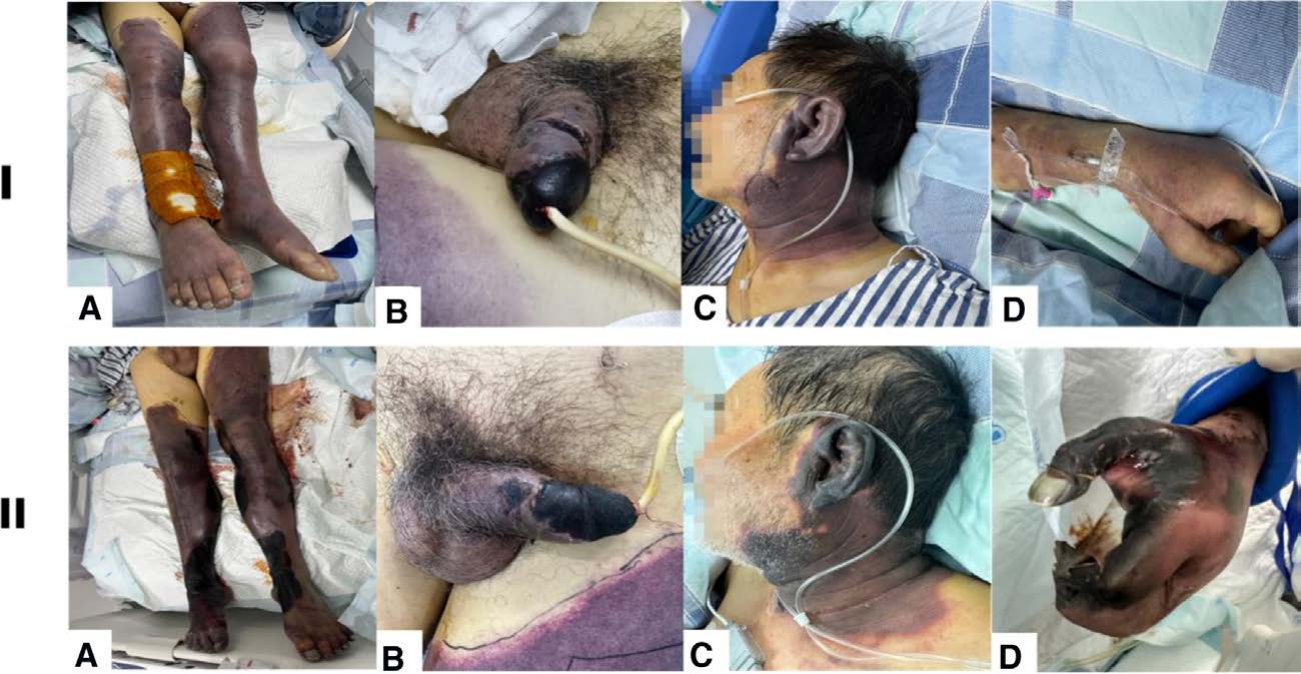
Clinical manifestations of the patient before and after treatment.

A 64-year-old man vaccinated against COVID-19 presented to the Emergency Department for swelling and pain in both lower extremities on March 14, 2023. The patient had a history of smoking for 30 years, splenectomy following trauma 7 years previously, and cough without medical attention for 2 months prior to the outbreak of COVID-19 in China. His main symptoms were swelling and pain in both lower limbs, followed by penile hyperemia, swelling, abnormal erection, and gradual blackening. He had been admitted to a local hospital with pulmonary embolism and deep vein thrombosis. Vascular ultrasonography revealed thrombosis of the left posterior tibial vein and bilateral calf intermuscular veins, and accessibility of the bilateral cavernous artery spectrum. Pulmonary artery computed tomography angiography showed a few pulmonary artery branch embolisms in the middle lobe of the right lung and lower lobes of both lungs, as well as multiple peripheral ground-glass opacities in both lungs (Fig. 1). A vena cava filter was implanted to trap the thrombus and prevent aggravation of the pulmonary embolism, as anticoagulation was contraindicated in patients with low platelet counts at that time (Table 1). However, the patient’s condition rapidly deteriorated, with a decrease in urine production. At the time of his admission to our hospital, the skin at the base of the thigh and below was purple and blackened, with multiple ruptured blisters. The dorsal arteries of both lower limbs were weak, the glans penis and both ears were blackened, the left forearm and left hand were slightly purple, and there was widespread purpura (Fig. 2). Ultrasonography showed a progressive increase in systemic thrombosis in the arteries and veins of both lower extremities, bilateral cavernous arteries, and small branches of the distal renal artery. Thrombosis in the renal artery caused acute renal failure, which consequently caused anuria and an increase in creatinine levels to 182.1 µmol/L. In addition, the patient had elevated lactate dehydrogenase levels, which suggested an underlying risk of muscle necrosis. Despite having stable circulation and oxygenation, he was immediately admitted to the intensive care unit as an extremely high-risk case and was treated with continuous renal replacement therapy. A review of the indicators revealed normal activity of coagulation factors II, V, VI to XII and protein S/C. However, the proportions of damaged and heterogenous red blood cells were 3% and 10%, respectively. Bone marrow aspiration revealed active granular hyperplasia with an increased neutrophil alkaline phosphatase score, active erythroid hyperplasia with a small amount of pathological hematopoiesis, and an average volume of megakaryocytes with poor platelet function. Further studies showed that the ADAMTS13 activity was 45.19%, excluding thrombotic thrombocytopenia purpura. Nevertheless, SARS-CoV-2 testing was positive without typical respiratory or other symptoms. Based on a combination of the patient’s clinical symptoms and auxiliary examinations, including low positivity for IgG anticardiolipin antibody (aCL) and lupus anticoagulant (LA), and high-titer IgA anti-β2GPI (Table 2), we were able to finally diagnose the patient with CAPS, COVID-19 (moderate), multiple vascular embolism, and multiple organ dysfunction.

**Table 1:** Changes in various indicators of the patient during the whole treatment.

	WBC	LY	M	NEU	HB	PLT	PT	INR	APTT	FIB	D2	ALT	TB	Cr	CRP	PCT	CK	LDH	ESR	IgG	IgA	IgM	C3	C4
	(4–10 × 10^9^/L)	(20–40%)	(0–12%)	(50–70%)	(130–175g/L)	(100–300 × 10^9^/L)	(12–15 s)	(0.91–1.22)	(30–43 s)	(2–4 g/L)	(≤0.5 mg/L)	(4.0–48 U/L)	(5.1–20.5 μmol/L)	(35–115 μmol/L)	(0.00–5.00 mg/L)	(<0.5 ng/mL)	(22–269 U/L)	(109–245 U/L)	(<15 mm/h)	(8–18 g/L)	(0.9–4.5 g/L)	(0.6–2.5 g/L)	(0.82–1.6 g/L)	(0.15–0.43 g/L)
A week ago	17.6	9.2	9.9	80.6	101	38	18.6	1.56	83.6	0.88	>20	24.9	16.3	81.0	203.88	–	2598.0	471.6	7	–	–	–	–	-
Admission	23.7	15.4	2.5	81.8	42	13	21.7	1.95	64.9	0.84	>20	144.5	16.0	182.1	111.87	3.27	8862.2	961.6	5	6.73	1.50	0.46	–	-
1st post PE	24.0	4.4	4.2	91.2	50	21	15.9	1.28	99.6	1.89	>20	55.6	20.0	84.9	50.00	1.94	2614.5	555.6	17	7.91	1.92	0.76	0.53	0.10
2nd post PE	26.2	1.8	3.1	94.9	77	32	14.1	1.10	55.1	2.86	>20	57.7	22.9	63.2	43.16	0.80	2486.7	693.0	34	8.77	2.35	1.14	0.64	0.09
3rd post PE	25.2	1.0	2.3	96.5	74	15	15.9	1.28	45.9	1.51	>20	58.3	36.5	102.3	44.40	–	2577.0	892.6	–	–	–	–	–	-
4th post PE	28.4	0.0	1.8	98.1	60	9	16.0	1.29	51.6	1.99	>20	61.6	44.4	133.7	48.50	0.93	2405.1	793.8	44	9.80	2.65	1.46	0.76	0.08
5th post PE	26.3	0.0	1.7	98.2	60	25	16.2	1.32	51.7	2.23	>20	64.3	43.1	110.4	50.20	0.79	–	–	64	10.23	2.89	1.33	0.83	0.10
6th post PE	18.3	0.4	2.5	97.0	63	33	15.4	1.24	51.5	2.79	20.0	46.8	26.6	114.5	49.90	0.58	612.6	525.2	81	9.70	2.66	1.28	0.79	0.11
7th post PE	19.5	0.4	2.8	96.7	75	37	15.0	1.20	54.8	3.26	>20	45.7	22.1	120.2	38.90	0.45	530.6	531.2	65	10.41	2.89	1.55	0.84	0.12
10th days	17.1	1.0	2.5	96.4	74	71	15.4	1.24	60.2	3.06	20.6	57.1	22.6	109.8	84.20	0.32	508.9	662.3	55	10.41	2.63	1.14	0.81	0.17
14th days	10.2	0.7	1.2	97.2	70	57	18.0	1.52	60.4	3.87	19.9	56.0	21.3	307.4	230.30	5.09	1153.5	664.6	73	9.80	2.23	0.58	0.67	0.22

ALT = alanine aminotransferase, aPTT = activated partial thromboplastin time, C3 = complement 3, C4 = complement C4, CK = creatine kinase, Cr = creatinine, CRP = C-reactive protein, D2 = D-dimer, ESR = erythrocyte sedimentation rate, FIB = fibrinogen, HB = hemoglobin, IgA = immunoglobulin A, IgG = immunoglobulin G, IgM = immunoglobulin M, INR = International Normalized Ratio, LDH = lactate dehydrogenase, LY = lymphocyte, M = monocyte, NEU = neutrophilic granulocyte percentage, PCT = procalcitonin, PE = plasmapheresis, PLT = platelet, PT = prothrombin time, TB = total bilirubin, WBC = white blood cell.

**Table 2:** Changes in indicators before and after treatment.

	Pre PE	Post PE	Reference Value
IgA aCL	<2.5	<2.5	N < 8.00; SP = 8.00 to 12; *P* ≥ 12.00 APLU/mL
IgM aCL	<5.00	<5.00	N < 8.00; SP = 8.00 to 12; *P* ≥ 12.00 MPLU/mL
IgG aCL	8.73	6.59	N < 8.00; SP = 8.00 to 12; *P* ≥ 12.00 GPLU/mL
IgA anti-β2GPI	67.8	70.5	0.0 to 20.0 U/mL
IgM anti-β2GPI	0.8	2.0	0.0 to 20.0 U/mL
IgG anti-β2GPI	16.6	17.9	0.0 to 20.0 U/mL
LA1	47.1	41.7	31.0 to 44.0 s
LA2	41.8	41.4	30.0 to 38.0 s
LA1/LA2	1.13	1.01	0.8 to 1.2
TNF-α	6.72	1.53	0.00 to 33.27 pg/mL
IFN-γ	4.00	6.73	0.00 to 20.36 pg/mL
IL-8	5.43	3.23	0.00 to 15.70 pg/mL
IL-2	2.01	1.65	0 to 6.64 pg/mL
IL-6	6.23	4.02	0 to 11.09 pg/mL
IL-4	5.96	3.99	0.00 to 8.37 pg/mL
IL-10	5.67	2.57	0 to 4.5 pg/mL
ANA	N	N	N
ANCA	N	N	N
SARS-CoV-2 testing	P	N	N

aCL = anticardiolipin antibody, ANA = antinuclear antibody, ANCA = anti-neutrophil cytoplasmic antibodies, anti-β2GPI = anti-β2-glycoprotein I antibody, COVID-19 NAT = coronavirus disease 2019 nucleic acid test, IFN-γ = interferon γ, IgA = immunoglobulin A, IgG = immunoglobulin G, IgM = immunoglobulin M, IL = interleukin, LA1 = lupus anticoagulant initial screening test, LA2 = lupus anticoagulant definitive test, N = negative, P = positive, SARS-CoV-2 = severe acute respiratory syndrome coronavirus-2, SP = suspicious positive, TNF-α = tumor necrosis factor α.

Unfortunately, the range of blackened and purple skin in the patient visibly expanded, and he developed transient unequal pupils, which implied a possibility of a transient ischemic attack. Because the patient had a high risk of bleeding, we administered small doses of unfractionated heparin (UFH), which were adjusted according to the activated partial thromboplastin time (aPTT). At the same time, plasmapheresis (PE) was selected to remove aPL, cytokines, and complements from the plasma within 24 hours after admission. The patient underwent 7 sessions, and approximately 2 L of plasma were processed in every session. The therapeutic regimen was simultaneously combined with 500 mg of intravenous methylprednisolone for 3 days, which was then reduced to 40 mg twice daily, and gradually transitioned to oral doses. Piperacillin sodium and tazobactam were used to fight the infection, and other symptomatic treatments were administered.

As the patient’s condition stabilized, a repeat ultrasound scan showed that thrombosis did not continue to increase, with a limited scope of necrosis, and microthrombosis in the kidneys disappeared with a gradual increase in urine production. Furthermore, coagulation function improved, and indicators of inflammation, such as C-reactive protein and procalcitonin, decreased. As hormonal effects may cause high white blood cell counts, platelet fluctuations were caused by the administration of continuous renal replacement therapy; therefore, we switched to an anticoagulant sodium citrate solution for in vitro anticoagulation, and the patient’s platelet count gradually increased as his condition improved. Following PE, IgG aCL and LA were negative, but the anti-β2GPI titer remained high. Additionally, irreversibly necrotic tissues, including the lower limbs, penis, left hand, and both ears, were complicated with infection. When inflammatory indicators again rose and both lower extremities emitted bad odor, we upgraded the patient’s treatment to meropenem to strengthen the anti-infection effects. We strongly recommended amputation to prevent severe infection or septic shock. The patient discharged himself from the hospital against the advice of his physician and died on the third day after discharge.

## 3. Discussion

In this report, the patient was diagnosed with CAPS with COVID-19, characterized by multiple thrombi and multiple organ dysfunction with acute kidney injury, necrosis of both lower limbs, left upper extremity, both ears, and penile gangrene. We controlled his clinical symptoms using active rescue treatments including anticoagulation, PE, glucocorticoid pulse therapy, antibiotics, and multi-organ function support. However, we were unable to save the necrotic tissue, and because the patient refused to undergo limb amputation and was discharged, he died of necrotic tissue infection.

The diagnosis of this patient is controversial. First, it is difficult to explain the multiple thrombi and organ dysfunction induced by only moderate COVID-19. Current research suggests that the pathogenesis of the thrombosis caused by COVID-19 and APS may involve common mechanisms, including endothelial cell activation and injury, platelet activation, complement activation, and release of neutrophil extracellular traps.^[4]^ In this patient, the COVID-19 infection may have played an essential role in the triggering of aPL, resulting in a life-threatening form, termed CAPS. According to the criteria for classifying the antiphospholipid syndrome, patients who do not show histopathological evidence of small vessel occlusion in at least 1 organ or tissue are diagnosed with probable CAPS in accordance with the criteria for the classification.^[5]^ However, this patient presented mainly with peripheral circulatory disorders and multi-organ necrosis, which was indirect evidence of small vessel occlusion. This case shows the need for caution and further assessment if IgA anti-β2GPI is the only positive aPL on enzyme-linked immunosorbent assay (ELISA).^[6]^ According to the revised Sapporo classification criteria of the APS in 2006,^[7]^ the laboratory criteria of APS require positive LA, IgM, IgG aCL, IgM, or IgG anti-β2GPI twice in an interval of at least 12 weeks. Diagnosing APS involves more than the classification criteria; e.g., non-criteria aPL such as IgA anti-β2GPI, which is highly specific for the identification of APS patients, contribute to better recognition of SNAPS. Anti-β2GPI binds to multiple receptors on the surface of platelets, leading to the activation of platelets and endothelium.^[8]^ It also activates the complement pathway which is significantly correlated with the coagulation pathways.^[9]^ IgA anti-β2GPI associated with thrombotic events^[10]^ are the most commonly observed aPL in patients with COVID-19 and were present in 28.8% (19 of 66) of critically ill patients.^[11]^ Before treatment, the patient in our case presented with low positivity for IgG in the aCL and LA assays. These levels may have been transiently elevated by infection or affected by acute thrombotic events. Muznay et al found that in APS due to systemic lupus erythematosus, the complete loss of aPL positivity after thrombosis was 41% for IgG aCL, 51% for IgM, 50% for IgA, and 20% for LA.^[12]^ aPLs may also be transient and occur in low titers in patients with APS and COVID-19. Therefore, it is suggested that there should be an interval of at least 12 weeks between a clinical event and the first positive laboratory test result. However, this patient did not provide us with an opportunity to implement this recommendation. In contrast, IgA anti-β2GPI was strongly positive, with values approximately 3 times the threshold. Our patient had widespread thrombosis, which severely consumed platelets and fibrinogen, prolonged prothrombin time-aPTT, and elevated D-dimer levels. Marked elevation of neutrophil and C-reactive protein levels indicated an acute form of systemic inflammation. Notably, this patient had only mildly elevated levels of interleukin-10, and other cytokines, including tumor necrosis factor α and interferon γ, were normal.

UFH, the mainstay treatment for CAPS, plays an important role in the inhibition of thrombin generation.^[13]^ However, there are no guidelines recommending therapeutic doses of UFH for patients with CAPS or COVID-19. Because of the high risk of bleeding, we empirically administered UFH to prolong aPTT by a factor of approximately 1.5. High-dose hormone pulse therapy was immediately administered, and 7 consecutive PE sessions were performed. The improvement in various indicators and thrombus suppression indicated the effectiveness of the treatment. However, necrotic organs and tissues could not be salvaged. If the patient had undergone amputation, his lifespan would have been extended.

This study had some limitations. First, the patient had a short hospital stay, so we were unable to observe changes in clinical manifestations and laboratory results throughout the course of the disease. Moreover, similar cases are rare, and we admitted only 1 patient, which limits the generalizability of our findings. Therefore, we recommend that more cases should be collected to enrich the research and improve our understanding of this disease.

In conclusion, complications from CAPS with COVID-19 can be severe, with rapid progression and a high mortality rate. Accurate diagnosis is crucial for appropriate and timely treatment, which can mitigate the severity of the disease and prevent devastating consequences. However, the pathogenesis of CAPS in patients with COVID-19 remains unclear. Future studies should investigate the possible relevant mechanisms to cure the disease.

## Author contributions

**Conceptualization:** Shanshan Jin, Jian Luo.

**Data curation:** Shiquan Wu.

**Formal analysis:** Shanshan Jin, Shiquan Wu, Jian Luo.

**Investigation:** Shanshan Jin, Bin Cai.

**Methodology:** Shanshan Jin, Bin Cai.

**Project administration:** Shanshan Jin, Shiquan Wu, Bin Cai.

**Resources:** Shiquan Wu, Bin Cai.

**Supervision:** Jian Luo.

**Validation:** Shanshan Jin, Shiquan Wu.

**Visualization:** Jian Luo.

**Writing – original draft:** Shanshan Jin.

**Writing – review & editing:** Jian Luo.
